# Supplementary material for: The interactome of intact mitochondria by cross-linking mass spectrometry provides evidence for coexisting respiratory supercomplexes
Source: Mol Cell Proteomics. 2017 Dec 8;17(2):216–32. doi: 10.1074/mcp.RA117.000470 (PMC5795388; doi:10.1074/mcp.RA117.000470)
Supplement: Supplemental Data [file supp_RA117.000470_133922_0_supp_23978_fzffwf.zip › spectra_annotation/mito_spectra_annotation/231-1-2-1-6-1.pdf]

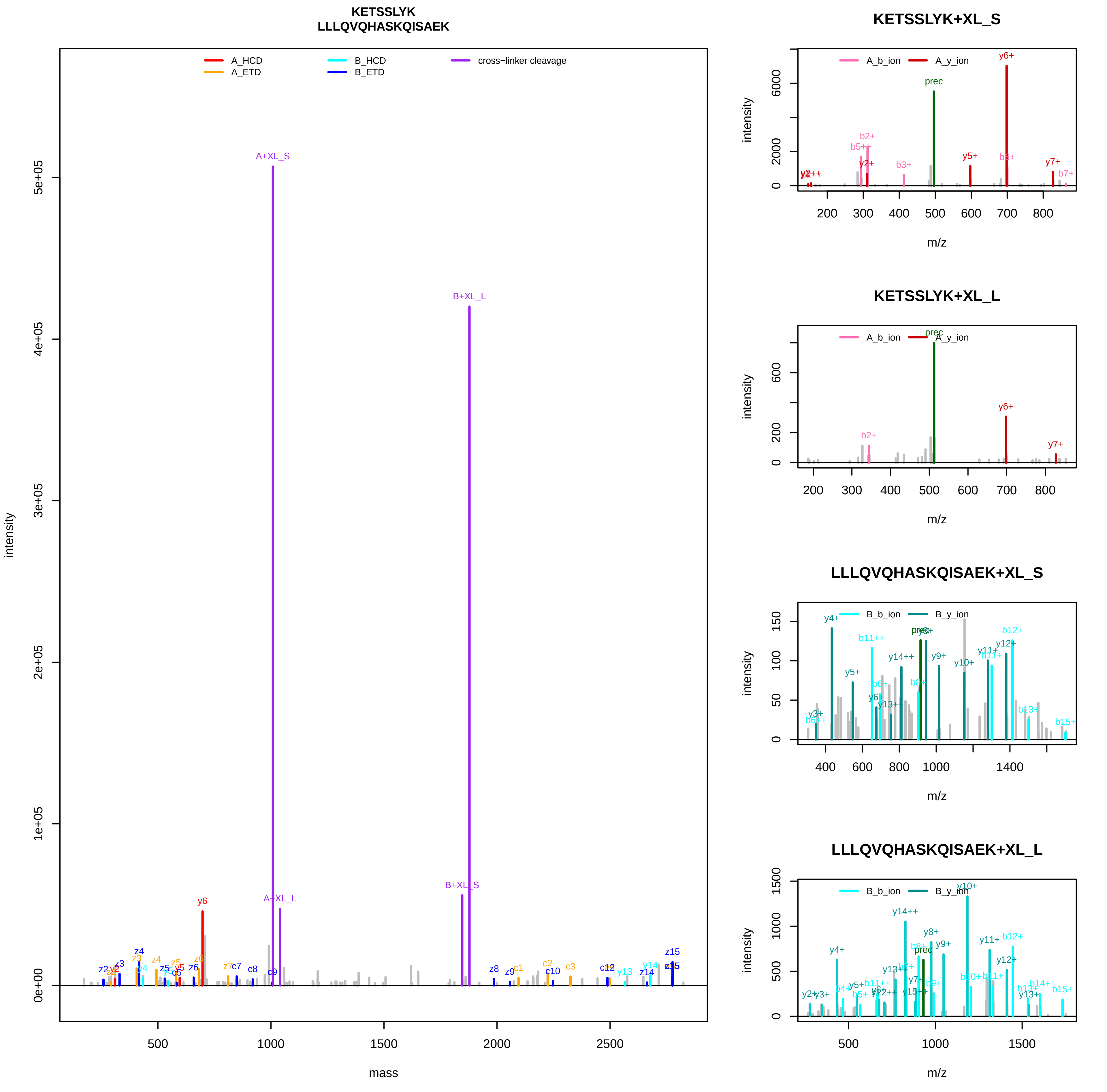
KETSSLYK
LLLQVQHASKQISAEK
A_HCD
A_ETD
B_HCD
B_ETD
cross−linker cleavage
A+XL_S
B+XL_L
A+XL_L
B+XL_S
y6
intensity
mass
0e+00
1e+05
2e+05
3e+05
4e+05
5e+05
500
1000
1500
2000
2500
KETSSLYK+XL_S
A_b_ion
A_y_ion
prec
y6+
b2+
b5++
y2+
b3+
y5+
b6+
y7+
b7+
m/z
KETSSLYK+XL_L
b2+
y6+
y7+
LLLQVQHASKQISAEK+XL_S
B_b_ion
B_y_ion
y4+
b11++
y14++
y9+
y10+
y11+
y12+
b12+
y5+
b13+
b15+
LLLQVQHASKQISAEK+XL_L
y10+
y14++
y8+
y4+
y11+
b12+
y12+
y9+
b15+
